# Eosinophilic Myocarditis: A Concise Review

**DOI:** 10.1007/s11886-024-02184-6

**Published:** 2025-01-23

**Authors:** Ashlee M. Asada, Rami Kahwash, Vincenzo Trovato

**Affiliations:** 1 Division of Internal Medicine, The Ohio State University, Columbus, OH USA; 2 Division of Cardiovascular Medicine, The Ohio State University, Columbus, OH USA; 3 Division of Heart and Vascular, Metrohealth Medical Center, 2500 Metrohealth Dr, Cleveland, OH 44109 USA

**Keywords:** Eosinophilic myocarditis, Hypereosinophilic syndrome, Eosinophilic granulomatosis with polyangiitis, Mepolizumab, Benralizumab

## Abstract

**Purpose of Review:**

Eosinophilic myocarditis (EM) is a rare and heterogeneous form of inflammatory heart disease that can present with a wide range of severity. Current literature is limited to case reports or small case series that outline the evaluation process, disease course, and the nonstandardized treatments trialed. This review aims to concisely summarize the current literature on EM including an update on maintenance therapy for refractory or recurrent disease.

**Recent Findings:**

In the last several years, several observational studies have reported the clinical benefit of mepolizumab and benralizumab in refractory EM.

**Summary:**

EM is a complex and heterogenous cause of inflammatory heart disease with a wide range of etiologies and presentations. Treatment of this disease has not been standardized as there are no large scale trials quantifying benefit of any specific therapy regimen. Targeted biologics show promise in observational studies; therefore, prospective studies are needed to quantify this benefit in EM.

## Introduction

Eosinophilic myocarditis (EM) is an inflammatory heart disease that can present with a wide range of clinical severity from asymptomatic to life-threatening. Though rare, EM has a significant in-hospital mortality rate as documented in prior systematic reviews of case studies [1–3]. EM is characterized by eosinophilic infiltration into cardiac tissue leading to inflammation and cardiac dysfunction. It is often associated with peripheral eosinophilia, however there have been case reports documenting EM without this typical finding [4]. Although there are numerous etiologies associated with EM, systematic reviews report idiopathic to be the most common. Other common etiologies associated with EM are hypersensitivity reactions, hypereosinophilic syndrome (HES), and eosinophilic granulomatosis with polyangiitis (EGPA) [1–3].

Although the exact mechanism of EM is not completely understood, the inflammatory response secondary to eosinophilic infiltration is well documented and is the target of treatment. However, there is no consensus on any EM-specific therapy given the lack of any large-scale clinical trials. Treatment regimens are chosen dependent upon the underlying etiology if known. Additionally, steroids are often used in the acute setting for their immunosuppressant effect. Current literature is limited regarding the disease course of EM and its management beyond the acute presentation, however a few case studies have reported a benefit of biologic therapy targeting interleukin (IL)-5 and its receptor in multitherapy refractory disease and in corticosteroid-dependent patients [5]. This review aims to concisely summarize the current literature on EM including an update on treatment regimens utilized in recent case studies.

## Pathophysiology

The inflammatory cellular damage secondary to eosinophilic infiltration characterizes the pathophysiology of EM. Histologically, the progression of disease is divided into three stages that may have overlapping features: acute necrosis, thrombosis, and fibrosis. The first acute necrosis stage is initiated by the cellular damage secondary to the degranulation of the eosinophils that have infiltrated into the myocardium. Increased expression of granulocyte/macrophage colony-stimulating factors, IL-3, and IL-5 receptors promote further degranulation which incites a cascade of mast cell recruitment and cellular damage through other mechanisms such as the release of major basic protein and eosinophilic cationic protein. Eosinophil degranulation also contributes to a hypercoagulable state through microvascular damage and activation of the coagulation system leading to the next thrombosis stage. The final fibrotic stage involves heart wall scar tissue formation and can also involve valvular structures [5].

## Epidemiology

EM is a rare disease though the true prevalence of EM is unknown given its heterogeneity in presentation as well as complexity of diagnosis. Of patients undergoing endomyocardial biopsy for suspected myocarditis, EM was reported in only 0.1% of cases [6]. In prior systematic studies EM was more prevalent in Caucasians with the mean age of 41 years in those with a histological diagnosis [1]. Two systematic reviews similarly showed that a systemic disorder was found to be associated as the underlying etiology in 64% and 71% of cases while the remaining were classified as idiopathic [2, 3].

## Etiology

Of the many underlying etiologies that can trigger eosinophilia, hypereosinophilic syndrome (HES), eosinophilic granulomatosis with polyangiitis (EGPA), and hypersensitivity reactions make up the majority [1–3]. A recent systematic review found that the most common etiology was idiopathic (28.8%) followed by EGPA (19.3%), drug-induced (13.1%), and HES (12.8%) with the remaining correlated to parasitic infection, malignancy-related, and vaccine-associated [3].

Drug-induced hypersensitivities have been linked to antibiotics namely minocycline and beta-lactams, clozapine, carbamazepine, indomethacin, diuretics, and vaccines such as tetanus toxoid and smallpox. Recent case reports have documented EM following COVID-19 mRNA vaccination. This exceedingly rare adverse event has mostly occurred in young males after receiving the second dose of these vaccines [7–11].

EGPA is a rare vasculitis that targets small to medium vasculature particularly in the pulmonary, cardiac, and renal systems. Cardiac involvement occurs in about 60% of patients and carries a poor prognosis with 50% mortality [12]. Diagnosis is based on the American College of Rheumatology criteria which is a composite score of specific clinical manifestations, peripheral eosinophil count, evidence of eosinophilic inflammation on biopsy, and presence of autoimmune markers such as cANCA.

HES is characterized by an absolute eosinophil count above 1.5 × 10^9^/L for more than six months with evidence of bone marrow, nervous system, or cardiac damage. Davies’ endomyocardial fibrosis and Loffler’s myocarditis are manifestations of HES. Etiology is often idiopathic (primary) but secondary HES typically is caused by hematological diseases.

## Clinical Presentation

Clinical presentations of EM are nonspecific much like other forms of myocarditis and can vary from asymptomatic to fulminant myocarditis. The most common symptoms are acute chest pain and dyspnea, mimicking acute coronary syndrome [3]. Syncope, palpitations, and other vague symptoms such as fever, myalgia, nausea, and fatigue have been reported. Notably patients can present with ventricular thrombi given the hypercoagulable state of this disease process [13, 14]. Other organ involvement can manifest as an atopic syndrome such as asthma, chronic rhinosinusitis, nasal polyposis, and skin involvement particularly in underlying EGPA or HES.

## Diagnosis

Diagnosis of eosinophilic myocarditis can be delayed as it is an uncommon form of myocarditis with nonspecific signs and symptoms that can present with a wide range of severity. In a recent systematic review, less than half of patients diagnosed with EM had pre-existing predilections such as asthma (31.8%), autoimmune disorder (9.1%), or atopic dermatitis (2.2%) [3]. High suspicion must be maintained as even those without peripheral eosinophilia or atopic symptoms on presentation can have eosinophilic cardiac infiltration. Brambatti et al. estimated that up to 25% of patients may not have peripheral eosinophil at time of evaluation [1]. In one small study, continued surveillance of cell counts noted that in 3 of 4 patients diagnosed with EM who initially had an eosinophil count of < 500/mm^3^ had an increase to > 500/mm^3^ after 7 to 12 days from onset [15].

As in other forms of myocarditis, laboratory findings can include elevated troponin, brain natriuretic peptide, and inflammatory markers such as erythrocyte sedimentation rate and C-reactive protein. Patients often have leukocytosis with severe eosinophilia but even fulminant cases of EM have been reported without this finding [4]. Nonspecific electrocardiogram changes may be present, most commonly tachyarrhythmias. Echocardiography may demonstrate nonspecific changes such as reduced ejection fraction, pericardial effusion, ventricular thrombi, and/or restrictive cardiomyopathy (in the fibrotic stage of EM) though no specific findings have been correlated. Subendocardial pattern of LGE can be seen on cardiac magnetic resonance imaging (CMR) which has emerged as a non-invasive modality in diagnosing EM. Though no large scale study exists validating CMR criteria alone for diagnosing EM, changes seen on CMR combined with other clinical clues may be enough to form a diagnosis. However, the gold-standard for diagnosis remains endomyocardial biopsy (EMB) which demonstrates myocardial necrosis with infiltration of eosinophils and lymphocytes [5].

A prior guideline for diagnosis proposed a strong suspicion for EM after ruling out acute myocardial infarction with the presence of the following: increased eosinophil count in peripheral blood (≥500/mm^3^), cardiac symptoms such as chest pain, dyspnea, or palpitations, elevated cardiac enzymes, electrocardiogram changes, and transient left ventricular wall thickening and abnormal wall motion on echocardiography [16]. More recently, a simplified diagnostic pathway proposed suspecting EM in the presence of symptoms of acute coronary syndrome or heart failure with normal coronary arteries and unexplained peripheral blood eosinophilia (>1.5 g/L) [2]. Both authors agree that though these findings can lead to the suspicion of EM, endomyocardial biopsy should be performed to confirm diagnosis by the presence of histological findings such as eosinophil infiltrates, degranulation of eosinophils, disappearance and fusion of cardiomyocytes, and interstitial edema and fibrosis via endomyocardial biopsy. Sampling error in the setting of patchy disease can lead to falsely negative results therefore adjunct CMR may be helpful to screen for EM and also guide EMB.

## Treatments

Along with guideline-directed medical therapy for those with reduced ejection fraction, specific treatment of EM is targeted at the underlying cause of eosinophilia; thus varies widely. In hypersensitivity reactions, identification and withdrawal of the implicated drug is required. Antihelminthic therapy is utilized for parasitic infections. Targeted tyrosine kinase inhibitors are used for the management of cases involving clonal myeloid disorders.

In EM associated with EGPA or HES, steroids are the mainstay treatment as up to 85% of cases are responsive to steroids [17]. However specific protocols used in these case studies are varied and there is a paucity of data leading to no clear consensus on optimal doses of pulse steroids during acute flares or taper regimen following clinical improvement. Treatment protocols are based on expert opinion and are extrapolated from the treatment of other inflammatory cardiomyopathies. Intravenous steroid bursts have been used inpatient if clinical presentation is severe or if rapid decline is seen. The tailoring of steroid bolus and taper dosing is often done on a case-by-case basis guided by the severity of initial symptoms, cardiac imaging, eosinophil counts, and clinical response to treatment.

Those that are refractory or develop side effects from long term use of steroids may be treated additionally with cytoreductive therapies or immunosuppressive agents such as cyclophosphamide, methotrexate, or azathioprine. Side effects for these agents include nausea and mucositis; other toxicities listed in Table 1. Case studies have shown the effectiveness of cyclophosphamide or methotrexate used for immunosuppression alone without steroids. For HES related EM, hydroxyurea or interferon-alpha has also been used. Other case studies have shown steroids in combination with azathioprine to be effective [5].

**Table 1:** Dosages and toxicities of second line agents used off-label to treat eosinophilic myocarditis

Drug	Dosage	Toxicity
Methotrexate	10–25 mg per week	Nausea, Mucositis, Blood, Teratogen, Liver, Lung
Cyclophosphamide	Oral: 50–150 mg/day IV: 500–2000 mg every 2 weeks	Nausea, Mucositis, Blood, Teratogen, Carcinogen, Bladder
Azathioprine	50–200 mg per day	Nausea, Mucositis, Blood, Teratogen, Carcinogen
Mepolizumab	Subcutaneous: 300 mg every 4 weeks	Hypersensitivity (anaphylaxis, angioedema, bronchospasm)

**Fig. 1:**
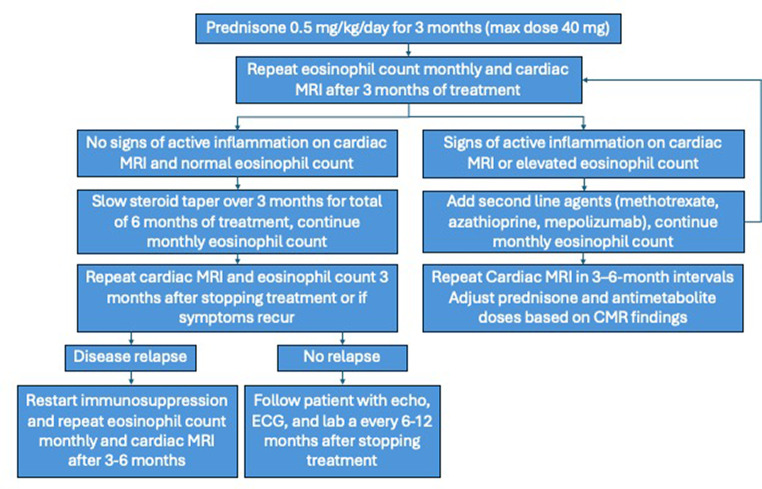
Proposed treatment algorithm for eosinophilic myocarditis

There has been an emerging use of other biologics for refractory disease that selectively target the inflammatory pathway involved in EM. The anti-interleukin (IL)-5 and the anti-IL-5 receptor monoclonal antibodies mepolizumab and benralizumab have been shown to be safe and effective corticosteroid-sparing agents in HES and EGPA in several trials [18–21]. They are FDA approved for the treatment of HES and EGPA and have been shown to lower eosinophil counts. However no large-scale studies have evaluated its use as targeted therapy for EM in the setting of these diseases. Several case studies of patients with EM in the setting of EGPA and HES have shown clinical benefit in the absence of significant adverse effects of these treatments (Table 2) [22, 23]. These case studies hold promise in a therapy that can potentially alter the disease course of patients that otherwise would develop chronic damage (fibrosis and restrictive cardiomyopathy) associated with ongoing inflammation, while avoiding the adverse effects associated with chronic corticosteroid use. Based on these studies, we propose a surveillance and treatment algorithm tailored to an individual patient’s disease course (Fig. 1). Overall, the approach to treatment should be made by shared decision making with the patient and should include counseling regarding potential side effects, toxicity risks, and need for monitoring. A multidisciplinary approach between cardiology and allergy/immunology experts is highly advised.

**Table 2:** Summary of recent case reports of patients with eosinophilic myocarditis treated with targeted IL-5/IL-5R biologics

Author (Year)	Relevant PMH/associated diagnosis	Cardiac imaging on presentation	Biologic treatment	Steroid adjustment following biologic initiation	Follow up cardiac imaging
Trovato et al. (2024) [22]	Patient 1: Asthma, HES Patient 2: Asthma, EGPA Patient 3: Allergic rhinitis, HES	Patient 1: Pericardial effusion with tamponade, CMR LVEF 31% Patient 2: Acute on chronic myocarditis on CMR, normal LVEF Patient 3: LVEF 45–50%	All patients: 300 mg mepolizumab monthly	Patient 1: Initial: 60 mg prednisone qd followed by taper 1 month: Successful taper off Patient 2: No steroid treatment Patient 3: Initial: 40 mg prednisone qd Following treatment: “low-dose” prednisone	Patient 1: CMR resolution of inflammation, stable LVEF Patient 2: Clinical improvement, stable CMR findings Patient 3: Stable functional capacity, LVEF 40–45%
Goyack et al. (2023) [23]	Eosinophilic Asthma	CMR LVEF 24%, RVEF 15% in the setting of fulminant cardiogenic shock requiring inotropic support	30 mg benralizumab every 2 months	Initial: 100 mg IV methylprednisolone daily x2 days, followed by taper 2 months: prednisone 5 mg qd	CMR LVEF 37%, RVEF 66%
Kodaka et al. (2022) [24]	Eosinophilic Asthma	TTE LVEF 41%	30 mg benralizumab every 2 months	Initial: 30 mg prednisolone qd followed by taper, recurrence of symptoms with prednisolone 2.5 mg qd 2 months: symptom resolution with maintenance 2.5 mg prednisolone qd	TTE LVEF 48%
Belfeki et al. (2021) [25]	Previously diagnosed EGPA	TTE: normal CMR: subepicardial enhancement areas on LGE	Prior to EM diagnosis: 1 g → 500 mg IV rituximab biannually Upon EM diagnosis: added 30 mg benralizumab monthly for 3 doses then every 2 months	Prior to EM diagnosis: 1 mg/kg prednisone qd in addition to rituximab 18 months following benralizumab: progressive tapering of prednisone	CMR: normalized cardiac signal with mild enhancement in apical segment
Truong et al. (2021) [26]	Previously diagnosed DRESS	TTE: LVEF 33%, moderately impaired RV systolic function	300 mg mepolizumab x2 doses (separated by 3 weeks) in addition to cyclophospha-mide	Initial: 250 mg methylprednisolone qd x3 days, followed by clinical worsening, thus 500 mg methylprednisolone qd x3 days 9 months: Successful taper off	Clinical improvement, no follow up cardiac imaging
Colantuono et al. (2020) [27]	EGPA	CMR: LVEF 40%, diffuse subendocardial inflammatory edema and fibrosis	30 mg benralizumab monthly for 3 doses then every 2 months	Initial: 1 mg/kg methylprednisolone qd x2 weeks Following benralizumab: 5 mg prednisone qd	TTE: LVEF 60% CMR: normal EF, improved edema, no changes of subendocardial fibrosis

*Abbreviations CMR* Cardiac magnetic resonance imaging, *DRESS* Drug reaction with eosinophilia and systemic symptoms, *EGPA* Eosinophilic granulomatosis with polyangiitis, *EM* Eosinphilic myocarditis, *HES* Hypereosinophilic syndrome, *IV* intravenous, *LGE* Late gadolinium enhancement, *LVEF* Left ventricular ejection fraction, *PMH* Past medical history, *Qd* daily, *RVEF* Right ventricular ejection fraction, *TTE* Transthoracic echocardiogram

## Conclusions

Eosinophilic myocarditis is a rare heterogeneous disease that requires high suspicion to diagnose. Although peripheral eosinophilia in the setting of myocarditis can raise suspicion for eosinophilic infiltration leading to cardiac dysfunction, this finding is not always present. Endomyocardial biopsy is the gold-standard for definitive diagnosis, while noninvasive CMR can provide further information such as the evaluation for ventricular thrombi and the commonly seen subendocardial pattern of LGE. CMR may also help guide tissue sampling avoiding sampling error of patchy disease. Given the rarity of EM, there are no large scale studies that outline a standardized treatment for eosinophilic myocarditis. Treatment is targeted at the underlying associated condition if known, such as hematologic malignancy, helminthic infection, or hypersensitivity. In primary systemic conditions such as EGPA or HES, steroids have shown benefit in the acute period. In refractory EM or in patient that are steroid-dependent, there is growing evidence of clinical benefit with the use of mepolizumab or benralizumab which targets the IL-5 and IL-5 receptors, respectively. Prospective studies are needed to quantify this observed benefit of clinical improvement in EM and also to standardize treatments including steroid protocol and the use of steroid-sparing biologics versus other immunosuppressant medications.

## Key References

- Techasatian, W., et al., *Eosinophilic myocarditis: systematic review.* Heart, 2024. **110**(10): p. 687–693.
  - **Systematic review of eosinophilic myocarditis case studies recently published.**
- Piccirillo, F., et al., *Eosinophilic Myocarditis: From Bench to Bedside.* Biomedicines, 2024. **12**(3).
  - **Review article of eosinophilic myocarditis.**
- Wechsler, M.E., Y. Fan, and P.A. Merkel, *Benralizumab versus Mepolizumab for Eosinophilic Granulomatosis with Polyangiitis. Reply.* N Engl J Med, 2024. **390**(20): p. 1940.
  - **Recent noninferiority clinical trial comparing benralizumab to mepolizumab for EGPA.**

## Data Availability

No datasets were generated or analysed during the current study.
